# Supplementary material for: Innovative behavior and organizational innovation climate among the Chinese clinical first-line nurses during the Omicron pandemic: The mediating roles of self-transcendence
Source: PLoS One. 2024 Jun 28;19(6):e0306109. doi: 10.1371/journal.pone.0306109 (PMC11213342; doi:10.1371/journal.pone.0306109)
Supplement: S1 Appendix — (DOCX) [file pone.0306109.s003.docx]

**Informed consent form**

Dear nurses,

Hope all is well with you!

First of all, thank you very much for taking time out of your busy schedule to fill out our questionnaire. The questionnaire consists of four sections. Please read the following contents carefully. If you have any questions or questions, please ask the researcher carefully.

The aims of this study are (1) to investigate the innovative behaviour, organizational innovation climate, and self-transcendence among Chinese clinical first-line nurses; (2) to examine the correlations among innovative behaviour, organizational innovation climate and self-transcendence; (3) to explore the mediating role of self-transcendence between innovative behaviour and organizational innovation climate; and (4) to provide a theoretical basis for constructing intervention measures to improve the innovative behaviour and ability of Chinese clinical first-line nurses, further improve the nursing quality and satisfaction during the Omicron pandemic and promote the development of clinical nursing

This study adopts questionnaire survey method. If you agree to participate in the study, the researchers will distribute questionnaires to you during the study. Please fill in the questionnaire truthfully according to your actual situation and ask the researchers in time if you don't understand the questions.

You voluntarily decide whether to participate in this study, and whether you participate in this study or not, you will not be affected in any way. All the information you provide is only used for this study, and it is kept safely, and will not be disclosed and made public to anyone without your permission. Thank you for your cooperation! During the research, if you have any questions, please feel free to consult the relevant personnel, and we will do our best to help you.

Wish: good health and all the best!

**Signature of researcher:**

**Date:**

The researcher has explained the research related matters to me, and I have made clear the purpose, process and significance of the research. I agree to participate in this research and sign here!

**Signature of participants:**

**Date:**

**1. First Section: The Demographic Characteristics Questionnaire (Note: Tick "√" in the box that best suits your situation.)**

| **Characteristics** | **Tick "√"** | **Characteristics** | **Tick "√"** |
| --- | --- | --- | --- |
| **Gender** |  | **Administrative position** |  |
| Male | □ | None | □ |
| Female | □ | Head nurse | □ |
| **Age (years)** |  | Head nurse of ward | □ |
| ≤ 25 | □ | Director of nursing department | □ |
| 26 ~ 30 | □ | **Hospital nature** |  |
| 31 ~ 35 | □ | Specialized hospital | □ |
| > 35 | □ | General hospital | □ |
| **Nursing age (years)** |  | **Whether is a specialist nurses** |  |
| ≤ 5 | □ | Yes | □ |
| 6 ~ 10 | □ | No | □ |
| > 10 | □ | **Whether is a clinical instructor** |  |
| **Marital status** |  | Yes | □ |
| Single | □ | No | □ |
| Married | □ | **Employment modality** |  |
| Divorced | □ | Enterprise system | □ |
| Widowed | □ | Contractual system | □ |
| **Number of children** |  | Labour dispatch system | □ |
| None | □ | **Income satisfaction** |  |
| 1 | □ | Dissatisfied | □ |
| ≥ 2 | □ | Average | □ |
| **Per capita monthly income (RMB)** |  | Satisfied | □ |
| < 3000 | □ | Very Satisfied | □ |
| 3000 ~ 4999 | □ | **Whether have ever applied for a nursing research project** |  |
| 5000 ~ 6999 | □ | Yes | □ |
| 7000 ~ 8999 | □ | No | □ |
| ≥ 9000 | □ | **Whether have ever published a paper** |  |
| **Education background** |  | Yes | □ |
| Technical secondary school | □ | No | □ |
| Junior college degree | □ | **Whether have attended a nursing research programme** |  |
| Bachelor degree | □ | Yes | □ |
| Master degree or above | □ | No | □ |
| **Technical title** |  |  |  |
| Nurse | □ |  |  |
| Nurse Practitioner | □ |  |  |
| Nurse-in-Charge | □ |  |  |
| Associate Nurse Practitioner | □ |  |  |
| Chief Nurse Practitioner | □ |  |  |

**2. Second Section: The Nurse Innovative Behaviour Scale (Note: Tick "√" in the box that best suits your situation.)**

| Items | Never | Less | Sometimes | Often | Frequently |
| --- | --- | --- | --- | --- | --- |
| **Generating ideas** | | | | | |
| 1.Generate the will to solve the problem. | □ | □ | □ | □ | □ |
| 2.Method of solving problems by using resource query. | □ | □ | □ | □ | □ |
| 3.Analyze the feasibility of solving problems in practical work. | □ | □ | □ | □ | □ |
| **Obtaining support** | | | | | |
| 4.Seek the approval, support and participation of colleagues or leaders. | □ | □ | □ | □ | □ |
| 5.Investigate the new method to get more information. | □ | □ | □ | □ | □ |
| 6.Seek financial support for new methods. | □ | □ | □ | □ | □ |
| 7.Formulate specific implementation plans for the new method. | □ | □ | □ | □ | □ |
| **Realizing ideas** | | | | | |
| 8.Apply the implementation scheme to work. | □ | □ | □ | □ | □ |
| 9.Revise the implementation plan and apply it in work. | □ | □ | □ | □ | □ |
| 10.Evaluate the effectiveness of new methods regularly. | □ | □ | □ | □ | □ |

**3. Third Section: The Nurse Organizational Innovation Climate Scale**

**(Note: Tick "√" in the box that best suits your situation.)**

| Items | Strongly disagree | Disagree | Not sure | Agree | Strongly agree |
| --- | --- | --- | --- | --- | --- |
| **Organizational innovation incentives** | | | | | |
| 1.The hospital/nursing department attaches importance to the cultivation of innovative talents. | □ | □ | □ | □ | □ |
| 2.The hospital/nursing department recognizes innovative nurses. | □ | □ | □ | □ | □ |
| 3.The hospital/nursing department encourages us to cooperate and innovate with other professionals. | □ | □ | □ | □ | □ |
| 4.The hospital/nursing department has a good consensus on innovation. | □ | □ | □ | □ | □ |
| 5.The hospital/nursing department set up an innovative overall goal/plan. | □ | □ | □ | □ | □ |
| 6.In the promotion evaluation, the hospital/nursing department attaches importance to our innovative achievements. | □ | □ | □ | □ | □ |
| 7.The hospital/nursing department organizes innovation award appraisal. | □ | □ | □ | □ | □ |
| **Resource supply** | | | | | |
| 8.The hospital/nursing department provides reasonable innovative financial support. | □ | □ | □ | □ | □ |
| 9.I have time to think and innovate. | □ | □ | □ | □ | □ |
| 10.The hospital/nursing department provides us with an effective way to declare patents or topics. | □ | □ | □ | □ | □ |
| 11.The hospital/nursing department provides abundant information resources for everyone to share. | □ | □ | □ | □ | □ |
| 12.The hospital/nursing department can provide effective help from our professionals. | □ | □ | □ | □ | □ |
| 13.The hospital/nursing department supports us to go out for exchange and study. | □ | □ | □ | □ | □ |
| **Management practices** | | | | | |
| 14.Department members can solve conflicts in the process of innovation through communication and coordination. | □ | □ | □ | □ | □ |
| 15.Division of labor and cooperation among department members in innovation activities. | □ | □ | □ | □ | □ |
| 16.Department members are willing to participate in the completion of innovative activities. | □ | □ | □ | □ | □ |
| 17.Department members can express their ideas without any worries. | □ | □ | □ | □ | □ |
| 18.The superior leaders encouraged us to express different opinions and viewpoints. | □ | □ | □ | □ | □ |
| 19.Department members will immediately reflect good ideas to their superiors. | □ | □ | □ | □ | □ |
| 20.The superior leaders are willing to listen to our ideas and suggestions. | □ | □ | □ | □ | □ |
| 21.The superior leaders will regularly ask about the progress of our innovative activities. | □ | □ | □ | □ | □ |

**4. Fourth Section: The Self-Transcendence Scale (Note: Tick "√" in the box that best suits your situation.)**

| Items (At the moment of my life,) | Non-conformance | Only some conformance | Some conformance | Very conformance |
| --- | --- | --- | --- | --- |
| 1.I am happy with my existing hobby or interest. | □ | □ | □ | □ |
| 2.When I am old, I can still accept myself.. | □ | □ | □ | □ |
| 3.I can still join the crowd or the community. | □ | □ | □ | □ |
| 4.I can adjust my present life well. | □ | □ | □ | □ |
| 5.I can accept and adjust to the changes in my physical function. | □ | □ | □ | □ |
| 6.I am willing to share my wisdom or experience with others. | □ | □ | □ | □ |
| 7.I can feel a lot of life truths from past experiences. | □ | □ | □ | □ |
| 8.I will help others in some ways. | □ | □ | □ | □ |
| 9.My interest in learning never stops. | □ | □ | □ | □ |
| 10.In the face of important events, I can think beyond the problem itself and solve it properly. | □ | □ | □ | □ |
| 11.I can accept that death is a part of life. | □ | □ | □ | □ |
| 12.I can find the meaning of life from my beliefs. | □ | □ | □ | □ |
| 13.I am willing to accept others' help when I need it. | □ | □ | □ | □ |
| 14.I enjoy the present pace of life. | □ | □ | □ | □ |
| 15.I can let go of my past gains and losses. | □ | □ | □ | □ |
