# Supplementary material for: Innovative behavior and organizational innovation climate among the Chinese clinical first-line nurses during the Omicron pandemic: The mediating roles of self-transcendence
Source: PLoS One. 2024 Jun 28;19(6):e0306109. doi: 10.1371/journal.pone.0306109 (PMC11213342; doi:10.1371/journal.pone.0306109)
Supplement: Attachment — Submitted filename: S1 File. Response to Reviewers.docx [file pone.0306109.s004.docx]

**Submission ID:** PONE-D-23-28215 R1

**Title:** Innovative behaviour and organizational innovation climate among Chinese clinical first-line nurses during the Omicron pandemic: The mediating roles of self-transcendence

February 22^th^, 2024

Dear academic editor and reviewers:

We would like to thank you for your efforts in reviewing our manuscript entitled “Innovative behaviour and organizational innovation climate among Chinese clinical first-line nurses during the Omicron pandemic: The mediating roles of self-transcendence”, and providing many helpful comments and suggestions, which will all prove invaluable in the revision and improvement of our paper, as well as in guiding our research in the future.

According to your nice suggestions, we have studied your comments point-by-point, and have made extensive corrections to our previous draft, the detailed corrections are listed below. All authors have approved the response letter and the revised version of the manuscript.

**Responds to the reviewers’ comments point-by-point:**

**Response to reviewer #1:**

Dear reviewer, we appreciate it very much for these good suggestion. Additionally, we also thank you for praising our manuscript is generally well-written. We are well aware that there are still some shortcomings in the manuscript. Therefore, in accordance with your suggestions, we have made the following revisions to our manuscript: .

**Q1:** “In the definition of innovative behavior the authors start form the definition of Scott et al [19] which is a wide ranging definition, and later on cite some of the specific literature, but some examples of what specifically are they are conceptualizing as innovative behavior in the Chinese clinical setup would be welcome.

**Response:** Thank you very much for your valuable comments, for this suggestion, we have made some revisions. We have further elaborated the definition of nurses' innovative behavior in China, which has the characteristics and cultural background of China, which is “Besides, Bao's [24] definition of nurses' innovative behavior is the most widely accepted in China: In order to promote health, prevent diseases and improve the quality of patients' care, nurses seek and develop new methods, technologies and working methods, and after obtaining the support of others, introduce and apply them to the behavioral process at work, including three stages: generating ideas, obtaining support and realizing ideas.” And all the changes have been marked in red in the revised draft, But it may not be so good. And our writing ability is limited, I hope you can be magnanimous.

**Q2:** “When laying out the Space Theory to express a concrete formula for innovative behavior (*Bi= f (Pi•Ei)*), it seems that the factor taken into consideration in this formula are wider than the ones taken in this study, p.e. Pi which considers personal internal needs of psychological factors can be considered much wider than the results expressed in the Nurse Innovative behavior scale and the Self-Transcendence Scale. The same goes for Ei and the Nurse Organizational Innovation Climate Scale. Therefore, a brief explanation of how the trim is justified would underscore this step, considering that measurement of such wide elements would be very hard.”

**Response:** Thank you very much for your valuable comments, for this suggestion, we have made some revisions. When we introduce the definitions of Innovative behaviour, organizational innovation climate and self-transcendence in the preface, we further elaborate their respective attribute characteristics. Of course, the range of variables included in Bi, Pi and Ei of the Space Theory is indeed very wide, and we also refer to the variables included in previous similar studies, which may be biased to some extent, and is also a deficiency of this study. And all the changes have been marked in red in the revised draft, But it may not be so good. And our writing ability is limited, I hope you can be magnanimous.

**Q3:** “Being that the sample is mostly women nurses, when discussing self-transcendence there is no consideration on gender and what could this complex concept mean to women or if some of the data could be disaggregated in this respect.”

**Response:** Thank you very much for your valuable comments, for this suggestion, we want to explain that: Because this study is a correlation study, plus the requirements of the number of words and the layout of the article. Therefore, in the data analysis, the three variables are not analyzed by single factor and multiple linear regression respectively, and only the status quo and reasons of the three variables are analyzed in the discussion part, and the influencing factors of the three variables are not analyzed separately. So, there is no consideration on gender and what could this complex concept mean to women when discussing self-transcendence. This is also a deficiency of this study, which is also described in the section "5.5 | Limitations". And all the changes have been marked in red in the revised draft, But it may not be so good. And our writing ability is limited, I hope you can be magnanimous.

Last but not least, we have studied your comments point by point, we tried our best to improve the manuscript and made some changes to the manuscript. These changes will not influence the content and framework of the paper. And here we did not list the changes in detail but marked in red in the revised paper. We appreciate for your warm work earnestly and hope that the correction will meet with approval. All authors have approved the response letter and the revised version of the manuscript.

And due to our writing ability, understanding and logic are limited and we look forward to your generosity. Due to the time limit of our project and the need to employ professional title, and this paper is very important to our team, so we sincerely hope that you can recommend our revised version of the manuscript to accept for publication in the journal *Plos One* as soon as possible. If you have any queries, please do not hesitate to contact me.

Thank you again for your valuable comments and suggestions. I'm looking forward to hearing from you soon in due course.

Best regards,

Zhangyi Wang, Master's degree, Nurse Practitioner, [283537548@qq.com,](mailto:283537548@qq.com,)

Nursing Department, Affiliated Hengyang Hospital of Hunan Normal University & Hengyang Central Hospital, Hengyang City, Hunan Province, 421001, China.
