# Supplementary material for: Innovative behavior and organizational innovation climate among the Chinese clinical first-line nurses during the Omicron pandemic: The mediating roles of self-transcendence
Source: PLoS One. 2024 Jun 28;19(6):e0306109. doi: 10.1371/journal.pone.0306109 (PMC11213342; doi:10.1371/journal.pone.0306109)
Supplement: Attachment — Submitted filename: S1 File. Response to Reviewers.docx [file pone.0306109.s005.docx]

**Submission ID:** PONE-D-23-28215 R2

**Title:** Innovative behavior and organizational innovation climate among the Chinese clinical first-line nurses during the Omicron pandemic: The mediating roles of self-transcendence

April 9^th^, 2024

Dear academic editor and reviewers:

We would like to thank you for your efforts in reviewing our revised manuscript entitled “Innovative behavior and organizational innovation climate among the Chinese clinical first-line nurses during the Omicron pandemic: The mediating roles of self-transcendence”, and providing many helpful comments and suggestions, which will all prove invaluable in the revision and improvement of our paper, as well as in guiding our research in the future.

According to your nice suggestions, we have studied your comments point-by-point, and have made extensive corrections to our previous draft, and all the changes have been marked in red in the revised draft, the detailed corrections are listed below. In order for reviewers and readers to better understand and read, we invited two nursing professors whose mother tongue is English and KetengEdit (www.ketengedit.com) to polish and revise the language of our article. All authors have approved the response letter and the revised version of the manuscript.

**Responds to the reviewers’ comments point-by-point:**

**Response to reviewer #1:**

Dear reviewer, we appreciate it very much for these good suggestion. Additionally, we also thank you for praising our manuscript is generally well-written. We are well aware that there are still some shortcomings in the manuscript. Therefore, in accordance with your suggestions, we have made the following revisions to our manuscript:

**Q1:** “I thank the authors for the changes made to the manuscript in attention to the previous observations. The manuscript has improved where it made those changes. I appreciate the attention paid to question 1., and the further details provided by Bao’s definition of nurses innovative behavior. In this regard, I consider the matter resolved.”

**Response:** For this, thank you very much for your praise.

**Q2:** “Concerning Q3, I think that not taking in consideration the matter of gender in such a sample is a limitation, but of course, the authors may legitimately choose to keep the study in such fashion and not consider analyzing the data considering differences between genders, however this could be explicitly stated in the limitations section.”

**Response:** Thank you very much for your valuable comments, for this suggestion, we have made some revisions. We also think that not taking in consideration the matter of gender in such a sample is a limitation in this study, which is also described in the section "5.5. Limitations", that is, “ Third, the study did not employ multiple linear regression analysis to examine the factors influencing innovative behavior, organizational innovation climate, and self-transcendence among nurses. Furthermore, the data analysis did not take gender differences in the sample into account. Additional investigation is needed to enhance the rigor of the design. It is recommended that future studies incorporate a greater number of clinical first-line nurses from various regions, delve into the factors that influence innovation behavior among these nurses, and account for gender disparities within the sample.” And all the changes have been marked in red in the revised draft, But it may not be so good. And our writing ability is limited, I hope you can be magnanimous.

**Q3:** “It is in Q2 where I found some difficulty to review the response of the authors, as they say “the range of variables included in Bi, Pi and Ei of the Space Theory is indeed very wide, and we also refer to the variables included in previous similar studies, which may be biased to some extent, and is also a deficiency of this study.” But when trying to trace how the other studies defined the Bi, Pi or Ei variables, I wasn’t able to read those study as they were mostly written in Chinese (language that I am unable to read). Therefore, I’m unable to follow the argument presented and therefore cannot judge the sufficiency of the changes made or the antecedents put forward, perhaps the argument should be made explicit in the text.”

**Response:** Thank you very much for your valuable comments, for this suggestion, we have made some revisions. First of all, we are sorry that this theory was put forward by Zhang Liming, a scholar in China, so we may not be able to read relevant English articles. Therefore, we further explain the argument of this theory, namely, “The theoretical framework of this study was Space Theory, proposed according to the field dynamics theory. According to the theory of field dynamics, the formula of innovation behavior is *Bi = f*·(*Si*), where innovation behavior (*Bi*) is a function of the innovation space, *Si*. According to this theory, innovation, as a social behavior, also has an innovative situation, and this situation is the innovative space. Therefore, another form of innovative behavior formula is finally obtained: *Bi = f* (*Pi*·*Ei*), where *Bi* stands for behavior (including innovative behavior), *Pi* stands for the person (including personal internal needs, internal psychological factors, and so forth), and *Ei* stands for the environment (including innovative environment and external environmental factors). Furthermore, it is important to note that the innovation subject *Pi* and the innovation environment *Ei* are interdependent variables that comprise *Si*. *Ei* can be understood as the sum of social and natural relations affecting *Bi* and *Si*, which can be regarded as the dynamic field of innovation behavior. In *Si*, *Pi* and *Ei* will interact. Based on the theories of space and field dynamics, the innovative behavior of nurses can be explained as a response to external factors that disrupt their usual working state. Nurses engage in innovative behavior to adapt to their environment, restore balance, and continue their work in nursing. During the Omicron pandemic, clinical first-line nurses must ensure the quality of care while facing greater work pressure, which may give birth to innovative behavior of nurses. Furthermore, this demonstrates how external factors impact the internal factors of nurses and promote their innovative behavior. In particular, combined internal and external factors can affect the innovative behavior of nurses, and may affect the innovative behavior of nurses through the intermediary role of internal factors.”

And further modify that conceptual framework diagram of Figure 1 in this study. What’s more, when we introduce the definitions of innovative behavior, organizational innovation climate and self-transcendence in the preface, we further elaborate their respective attribute characteristics. And all the changes have been marked in red in the revised draft, but it may not be so good. And our writing ability is limited, I hope you can be magnanimous.

**Q4:** “Consider correcting the following: pg. 25 “Innovation behaviour.among clinical first-line nurses.”

**Response:** Thank you very much for your valuable comments, for this suggestion, we have changed it to “Innovation behaviour among clinical first-line nurses.”

**Response to reviewer #2:**

Dear reviewer, we appreciate it very much for these good suggestion. We are well aware that there are still some shortcomings in the manuscript. Therefore, in accordance with your suggestions, we have made the following revisions to our manuscript:

**Q1:** “1. Samples: The mere mention of "seven hospitals in China" without details on their location, size, or administrative level significantly undermines the study's generalizability. Moreover, while nurse characteristics are described, information on the hospitals is notably absent but essential.”

**Response:** Thank you very much for your valuable comments, for this suggestion, we have made some revisions. We have described and supplemented the hospital information in the section of “3.2. Participants and sample” and the section of “4.1. Demographic characteristics”, that is, “Convenience sampling was used to recruit the nurses from seven tertiary grade-A hospitals during the Omicron pandemic, which are all located in a municipality directly under the central government in Northern China, with more than 1,500 beds and 2,000 employees.” “A total of 1,073 Chinese clinical first-line nurses were recruited to participate in the survey from seven tertiary grade-A hospitals located in a municipality directly under the central government in Northern China, with more than 1,500 beds and 2,000 employees. Among them, there are five general and two specialty hospitals.”, respectively. But it may not be so complete, which is also a deficiency of this study. Moreover, due to the blind review of the *Plos One* and the limitation of hospital privacy, we failed to describe the specific names and cities of seven hospitals, but only described their nature, size, number of hospital beds and number of employees, etc.We also think the sample of this study is a limitation in this study, therefore, it is also described in the section "5.5. Limitations", that is, “First, the study was conducted using a convenience sampling method. Only 1,058 Chinese clinical first-line nurses were enrolled from seven tertiary grade-A hospitals, which were all located in a municipality under the central government in Northern China. This may lead to unrepresentative samples and one-sided, non-generalized, or limited results. Therefore, random, multi-center research with a large sample should be conducted in the future.” And all the changes have been marked in red in the revised draft, but it may not be so good. And our writing ability is limited, I hope you can be magnanimous.

**Q2:** “2. Sample Size: The method described for calculating sample size is incorrectly attributed to Kendall's "Multivariate Analysis," confusing the number of items with variables. The approach is inappropriate for this study's aims, specifically concerning the precision of correlation coefficient estimation. Moreover, the cited page range (251-306) does not exist in Kendall's work, which casts doubt on the draft's accuracy.”

**Response:** Thank you very much for your valuable comments, for this suggestion, we have made some revisions. We have changed “Kendall” to “G*Power 3.1.9.7 software”, that is, “G*Power 3.1.9.7 software was used to calculate the minimum sample size required for correlation analysis (α error probability was 0.05, and test validity was 80%). According to related research, the average correlation coefficient among innovative behavior, organizational innovation climate, and self-transcendence is 0.17, and the sample size is calculated using a *t*-test. In G*Power 3.1.9.7 software, the *t*-tests method was selected, and "Correlation: Point biserial model" was selected, and then the parameter effect size |ρ| = 0.17, α = 0.05, β = 0.8 were set, and finally clicked "Calculate" to calculate the total sample size = 266, the minimum sample size of this study. In the end, 1, 058 participants were included in this study.” And all the changes have been marked in red in the revised draft, But it may not be so good. And our writing ability is limited, I hope you can be magnanimous.

**Q3:** “3. Theoretical Foundation: Although leveraging field theory is intriguing, its application to the authors' mediating variable model is inadequately justified and discussed.”

**Response:** Thank you very much for your valuable comments, for this suggestion, we have made some revisions. We further explain the argument of this theory, namely, “The theoretical framework of this study was Space Theory, proposed according to the field dynamics theory. According to the theory of field dynamics, the formula of innovation behavior is *Bi = f*·(*Si*), where innovation behavior (*Bi*) is a function of the innovation space, *Si*. According to this theory, innovation, as a social behavior, also has an innovative situation, and this situation is the innovative space. Therefore, another form of innovative behavior formula is finally obtained: *Bi = f* (*Pi*·*Ei*), where *Bi* stands for behavior (including innovative behavior), *Pi* stands for the person (including personal internal needs, internal psychological factors, and so forth), and *Ei* stands for the environment (including innovative environment and external environmental factors). Furthermore, it is important to note that the innovation subject *Pi* and the innovation environment *Ei* are interdependent variables that comprise *Si*. *Ei* can be understood as the sum of social and natural relations affecting *Bi* and *Si*, which can be regarded as the dynamic field of innovation behavior. In *Si*, *Pi* and *Ei* will interact. Based on the theories of space and field dynamics, the innovative behavior of nurses can be explained as a response to external factors that disrupt their usual working state. Nurses engage in innovative behavior to adapt to their environment, restore balance, and continue their work in nursing. During the Omicron pandemic, clinical first-line nurses must ensure the quality of care while facing greater work pressure, which may give birth to innovative behavior of nurses. Furthermore, this demonstrates how external factors impact the internal factors of nurses and promote their innovative behavior. In particular, combined internal and external factors can affect the innovative behavior of nurses, and may affect the innovative behavior of nurses through the intermediary role of internal factors.”

And the conceptual framework and theoretical basis of Figure 1 in this study are further modified and explained. What’s more, when we introduce the definitions of innovative behavior, organizational innovation climate and self-transcendence in the preface, we further elaborate their respective attribute characteristics. And all the changes have been marked in red in the revised draft, but it may not be so good. And our writing ability is limited, I hope you can be magnanimous.

**Q4:** “Statistical Model: The core model may be overlooked potential confounders other than "organizational innovation climate" and "self-transcendence" that might significantly affect "innovative behavior".”

**Response:** Thank you very much for your valuable comments, for this suggestion, we also agree with the limitations of the core statistical model. Therefore, it is also described in the section "5.5. Limitations", that is, “Fourth, the statistical model may have overlooked potential confounders other than "organizational innovation climate" and "self-transcendence" that might significantly affect "innovative behavior." Therefore, more accurate and scientific statistical models must be adopted in future studies, along with more factors that may significantly affect innovation behavior.” And all the changes have been marked in red in the revised draft, but it may not be so good. And our writing ability is limited, I hope you can be magnanimous.

**Q5:** “Table 1: The inclusion of P-values and the unclear meaning of "Z/*H*" are problematic. Descriptive information should not intertwine with inferential statistics, contrary to what STROBE guidelines advocate (Item 14, Explanation).”

**Response:** Thank you very much for your valuable comments, for this suggestion, we have made some revisions in Table 1. Because the aims of this study are (1) to investigate the innovative behavior, organizational innovation climate, and self-transcendence among the Chinese clinical first-line nurses, (2) to examine the correlations among innovative behavior, organizational innovation climate, and self-transcendence, (3) to explore the mediating role of self-transcendence between innovative behavior and organizational innovation climate. In this study, we didn't compare the differences of innovative behavior of nurses with different demographic characteristics, and the results of univariate analysis of innovative behaviour are also not included in the subsequent section “5. Discussion”. Maybe this part is of little significance to the structure of the whole article, and the limitation of layout and number of tables, so the original section “4.3. Univariate analysis of innovative behaviour” is deleted and Table 1 is changed to table of descriptive information after full discussion among the our team members. And all the changes have been marked in red in the revised draft, But it may not be so good. And our writing ability is limited, I hope you can be magnanimous.

**Q6:** “6. Mediating Effect of Self-Transcendence: This section and Figure 2 inadequately report the direct effects between "organizational innovation climate" and "innovative behavior," requiring a clearer presentation in both figure and table formats.”

**Response:** Thank you very much for your valuable comments, for this suggestion, we have made some revisions in the section of “4.5. Mediating effect of self-transcendence between innovative behavior and organizational innovation climate” and Figure 2, which can clearly reflect the direct effect, indirect effect and total effect of each variable and the correlation between variables. And all the changes have been marked in red in the revised draft, But it may not be so good. And our writing ability is limited, I hope you can be magnanimous.

**Q7:** “1. References: Several citations are incomplete or incorrect, such as the nonexistent "Review of Principles of Topological Psychology" and the misquoted page range in Kendall's work. Additionally, the "PROCESS" program used for mediation analysis is not cited. A thorough review and correction of the reference list are recommended.”

**Response:** Thank you very much for your valuable comments, for this suggestion, we have made some revisions. We have replaced the above two references again. And the "PROCESS" program used for mediation analysis is cited in the manuscript. What’s more, we have a thorough review and correction of the reference list. And all the changes have been marked in red in the revised draft, But it may not be so good. And our writing ability is limited, I hope you can be magnanimous.

Last but not least, we have studied your comments point by point, we tried our best to improve the manuscript and made some changes to the manuscript. These changes will not influence the content and framework of the paper. And here we did not list the changes in detail but marked in red in the revised paper. We appreciate for your warm work earnestly and hope that the correction will meet with approval. All authors have approved the response letter and the revised version of the manuscript.

And due to our writing ability, understanding and logic are limited and we look forward to your generosity. Due to the time limit of our project and the need to employ professional title, and this paper is very important to our team, so we sincerely hope that you can recommend our revised version of the manuscript to accept for publication in the journal *Plos One* as soon as possible. If you have any queries, please do not hesitate to contact me.

Thank you again for your valuable comments and suggestions. I'm looking forward to hearing from you soon in due course.

Best regards,

Zhangyi Wang, Master's degree, Nurse Practitioner, [283537548@qq.com,](mailto:283537548@qq.com,)

Nursing Department, Affiliated Hengyang Hospital of Hunan Normal University & Hengyang Central Hospital, Hengyang City, Hunan Province, 421001, China.
