# Supplementary material for: Innovative behavior and organizational innovation climate among the Chinese clinical first-line nurses during the Omicron pandemic: The mediating roles of self-transcendence
Source: PLoS One. 2024 Jun 28;19(6):e0306109. doi: 10.1371/journal.pone.0306109 (PMC11213342; doi:10.1371/journal.pone.0306109)
Supplement: Attachment — Submitted filename: S1 File. Response to Reviewers.docx [file pone.0306109.s006.docx]

**Submission ID:** PONE-D-23-28215 R3

**Title:** Innovative behavior and organizational innovation climate among the Chinese clinical first-line nurses during the Omicron pandemic: The mediating roles of self-transcendence

May 9^th^, 2024

Dear academic editor and reviewers:

We would like to thank you for your efforts in reviewing our revised manuscript entitled “Innovative behavior and organizational innovation climate among the Chinese clinical first-line nurses during the Omicron pandemic: The mediating roles of self-transcendence”, and providing many helpful comments and suggestions, which will all prove invaluable in the revision and improvement of our paper, as well as in guiding our research in the future.

According to your nice suggestions, we have studied your comments point-by-point, and have made extensive corrections to our previous draft, and all the changes have been marked in red in the revised draft, the detailed corrections are listed below. In order for reviewers and readers to better understand and read, we invited two nursing professors whose mother tongue is English and KetengEdit (www.ketengedit.com) to polish and revise the language of our article. All authors have approved the response letter and the revised version of the manuscript.

**Responds to the reviewers’ comments point-by-point:**

**Response to reviewer #2:**

Dear reviewer, we appreciate it very much for these good suggestion. We are well aware that there are still some shortcomings in the manuscript. Therefore, in accordance with your suggestions, we have made the following revisions to our manuscript:

**Q1:** “1. The introduction of the manuscript is verbose. The authors should consider the following suggestions:

-First, the background information provided is excessively detailed. The detailed descriptions of COVID-19, particularly the Omicron variant, are well-known and seem unnecessary in this manuscript. Since the intended audience of this study is likely already familiar with this information, it could be omitted. Instead, briefly state why the characteristics of COVID-19 and the Omicron variant are relevant in the context of this study.

-Second, there is repetitive information. The necessity for innovation in nursing and quality improvement is mentioned multiple times without each mention providing new information. While it is important to emphasize key points, repetition can scatter the reader's attention. Avoiding repetition and stating each point clearly once would enhance the clarity of the text.

-Lastly, unnecessary detailed descriptions should be avoided. Several cited studies are described in excessive detail. Unless these details are directly necessary for understanding this research, it would be more appropriate to succinctly state the main findings and how these studies contribute to the discussion of the paper. This approach would make the text more focused and easier to read.”

**Response:** Thank you very much for your valuable comments, for this suggestion, we have made major revisions, that is:

(1) First, we have simplified the “Introduction” as a whole, from 1659 words to 1223 words.We have reorganized the section of “Introduction” and divided it into several paragraphs, which makes the overall structure more layered and logical. In order to make it easier to understand and read, we added the subtitle "1.1. Literature review" in the section of “Introduction”. We have also deleted the detailed descriptions of COVID-19, and stated why the characteristics of COVID-19 and the Omicron variant are relevant in the context of this study, that is, “Compared to Delta or SARS-CoV2, Omicron has more vital transmission ability, faster transmission speed, shorter incubation period, and rapid disease progression. These characteristics have had a profound negative influence on public health and have significantly impacted patients and clinical first-line nurses. By the end of 2022, many nurses in China were deployed to the frontlines of clinical care to assist in managing and containing the Omicron pandemic. However, clinical first-line nurses faced a greater risk of contracting Omicron than the general population. Not only do they have to endure the demanding workload brought about by the pandemic, but they also need to continuously innovate traditional nursing models and methods in clinical practice and enhance innovative awareness and ability to adapt to the pandemic-induced changes. And many studies have demonstrated that clinical first-line nurses were known to be at risk for depression, anxiety, fear, post-traumatic stress disorder, and enthusiasm for innovation to varying degrees during the Omicron pandemic, significantly higher than the SARS pandemic or general population. Studies have shown that, improving nurses' innovative behavior is a robust measure for addressing resource scarcity, heavy clinical nursing burden, and managing the Omicron pandemic challenges.”

(2) Second, we have deleted repetitive information of necessity for innovation in nursing and quality improvement. In the first paragraph of the “Introduction”, the importance of nurses' innovative behavior is put forward. And put forward the limitations of existing research and the importance of this study, that is, “However, few research were on the innovative behavior of clinical first-line nurses and its related correlation study during the Omicron pandemic in China. Therefore, it is essential to investigate the innovative behavior of clinical first-line nurses and explore the relationships between other related variables.”

(3) Lastly, we have succinctly state the main findings and how these studies contribute to the discussion of the paper. For several cited studies are described in excessive detail, we deleted some repetitive cited references, and the description was simplified. And the cited references in this study were reduced from 70 to 55, the order of all references has also been updated in the "References" section. In the third and fourth paragraphs of the “Introduction”, we put forward the general argument of the relationship between nurses' innovative behavior, organizational innovation climate and self-transcendence, and made it bold. In this way, we can clearly understand the research status and unresolved key issues of this study, which makes the overall structure more layered and logical.

And all the changes have been marked in red in the revised draft.

**Q2:** “2. There are some discrepancies between the statistical analysis design and the sample size calculations. In order to enhance the clarity, completeness, and scientific rigor of the study, it would be beneficial to consider the following points:

Analysis of Mediating Effects: The purpose of this study is not only to explore the correlation between innovative behavior, organizational innovation climate, and self-transcendence, but also to investigate the mediating role of self-transcendence between innovative behavior and organizational innovation climate. Given this objective, the sample size calculation should include considerations specific to mediation analysis. This type of analysis requires a sample size that adequately addresses the relationships among the independent, mediating, and dependent variables, taking into account the expected effect sizes of the paths involved. The description of sample size calculations in the current manuscript does not appear to meet these requirements. The following book is recommended as a reference: Hayes AF. Introduction to Mediation, Moderation, and Conditional Process Analysis: A Regression-Based Approach. 3rd ed. New York. : Guilford Publications; 2022 Jan 24. ISBN 9781462549030.

-Model Selection for Sample Size Calculation: The manuscript mentions that a "point biserial model" was used to calculate the sample size. However, this model is usually employed when one variable is binary and the other is continuous. In a mediation analysis, where usually all variables involved are continuous, the choice of this model may not be appropriate. A more detailed explanation of why this model was chosen and its suitability for the reported analysis is needed.

-Sample size discrepancy: the manuscript states that the "point biserial model" was used to calculate a minimum sample size of 266 participants, but the study included 1,058 participants. This large difference in numbers raises questions about the rationale for including such a large sample. While the large sample size may be justified by the requirement for a comprehensive mediation analysis, the manuscript currently lacks an explanation for this discrepancy. Clarifying why 1,058 participants were necessary would greatly enhance the transparency and understanding of the study design, especially in relation to the mediation analysis. understanding of the study design, especially in relation to the mediation analysis.

-Consistency of reporting: In my previous review I noted that the reporting of descriptive statistics should avoid mixing information from inferential statistics, and that this has been noted in STROBE as well. The authors accept this point and exclude the reporting of p-values from the summary of descriptive statistics results as seen in Table 1. If this is the case, reference to inferential statistical methods for between-group comparisons should also no longer be included, as it may confuse the reader or imply analytical methods not employed in the study.”

**Response:** Thank you very much for your valuable comments, for this suggestion, we have made some major revisions, that is:

(1) First, by studying this book “Hayes AF. Introduction to Mediation, Moderation, and Conditional Process Analysis: A Regression-Based Approach. 3rd ed. New York. : Guilford Publications; 2022”, we have changed “*t*-tests” to “*F*-tests” of G*Power 3.1.9.7 software, that is, “G*Power 3.1.9.7 software was used to calculate the minimum sample size required for *F*-tests [ α error probability was 0.05, and power (1 - β error probability) was 95% ]. Because all variables involved of this study are continuous, therefore, the *F-*tests method was selected in G*Power 3.1.9.7 software, “Linear multiple regression: Fixed model, *R*^2^ deviation from zero” of statistical test was selected, “A priori: Compute required sample size - given α, power, and effect size” of type of power analysis was selected, and then the parameter effect size *f*²= 0.15, α = 0.05, 1 - β = 0.95 were set, and finally clicked "Calculate" to calculate the total sample size = 107, the minimum sample size of this study.”

(2) Second, we clarified why 1,058 participants were necessary would greatly enhance the transparency and understanding of the study design, especially in relation to the mediation analysis, that is, “Considering the hospital and clinical realistic environment, 1,058 participants were included in this study in the end. And “Post hoc: Compute achieved power - given α, sample size, and effect size” of type of power analysis was selected. And then the parameter effect size f²= 0.15, α = 0.05, total sample size = 1058 were set, and finally clicked "Calculate" to calculate the 1 - β = 1.00, which was greater than the original 1 - β of 0.95 and indicating that the sample size of the study meets the sample size required for the mediation analysis. In addition, participants were recruited from seven tertiary grade-A hospitals of Tianjin city in Northern China through online questionnaires, and collected more questionnaires to ensure the generalization and universality of the research results, so as to minimize the deviation of the results caused by geography and hospital nature. In this way, included 1,058 participants were necessary and would greatly enhance the transparency and understanding of the study design, especially in relation to the mediation analysis. ”

(3) Lastly, we have deleted inferential statistical methods for between-group comparisons in the section of “3.4. Statistical analysis”

And all the changes have been marked in red in the revised draft.

**Q3:** “3.1. Research Design and Setting" should include detailed information about the setting. It is necessary to specify the time period (year and month) during which the study was conducted. Rather than merely stating 'China,' providing a more detailed location, such as the province or city, would be beneficial. Additionally, references to STROBE should be concise, only noting whether this report of the study is STROBE compliant.”

**Response:** Thank you very much for your valuable comments, for this suggestion, we have made some revisions, that is, “ This study used a descriptive cross-sectional design and was conducted of Tianjin city in Northern China from March 2022 to February 2023. The quality reporting of the study adhered to the STROBE Checklist (see S1).”

And all the changes have been marked in red in the revised draft.

**Q4:** “It is recommended to divide the section "3.2. Participants and Sample" into two different sections: “Participants” and “Sampling Methods and Sample Size Calculation.” In the “Participants” subsection, the inclusion and exclusion criteria should be detailed. In the “Sampling Methods and Sample Size Calculation” subsection, the focus should be on the procedures used to recruit participants and how the sample size was calculated. This restructuring will enhance the logical flow of the sections and make the information more organized and accessible.”

**Response:** Thank you very much for your valuable comments, for this suggestion, we have made some revisions, that is:

**“3.2. Participants and sample**

**3.2.1. Participants**

Convenience sampling was used to recruit the nurses from seven tertiary grade-A hospitals during the Omicron pandemic, which are all located in Tianjin city of Northern China, with more than 1,500 beds and 2,000 employees. Respondents met the following criteria—inclusion criteria: (1) obtained a nurse's professional qualification and register certificates; (2) Worked in the first-line during the Omicron pandemic for ≥ 1 month. Exclusion criteria: (1) intern nurses, (2) advanced training, rotation, and regular training nurses, and (3) nurses who were not on duty during the investigation period. Written informed consents were taken from the nurses who volunteered to participate in the study.

**3.2.2. Sampling methods and sample size calculation**

Participants were recruited from seven tertiary grade-A hospitals of Tianjin city in Northern China from March 2022 to February 2023. The investigation was conducted with the hospitals' prior approval, focusing on individual departments and with the assistance of the head nurse in each department for distribution. We used the unified instruction language to explain the essential information to the participants, including the purpose, significance, and confidentiality of this study, and the participants were investigated face-to-face using an online questionnaire. The questionnaires underwent quality control through several measures: (1) each micro-signal can be filled only once, after being authorized by the system settings; (2) the participants are required to answer all the questions; (3) questionnaires with completion time less than one minute were excluded; (4) questionnaires with obvious contradictory answers were excluded; (5) questionnaires with obvious inconsistent personal information of the respondents were excluded. Finally, 1,058 valid questionnaires were collected, and the effective recovery rate was 96.7%.

G*Power 3.1.9.7 software was used to calculate the minimum sample size required for F-tests [ α error probability was 0.05, and power (1 - β error probability) was 95% ]. Because all variables involved of this study are continuous, therefore, the F-tests method was selected in G*Power 3.1.9.7 software, “Linear multiple regression: Fixed model, R2 deviation from zero” of statistical test was selected, “A priori: Compute required sample size - given α, power, and effect size” of type of power analysis was selected, and then the parameter effect size *f*²= 0.15, α = 0.05, 1 - β = 0.95 were set, and finally clicked "Calculate" to calculate the total sample size = 107, the minimum sample size of this study.

Considering the hospital and clinical realistic environment, 1,058 participants were included in this study in the end. And “Post hoc: Compute achieved power - given α, sample size, and effect size” of type of power analysis was selected. And then the parameter effect size *f*²= 0.15, α = 0.05, total sample size = 1058 were set, and finally clicked "Calculate" to calculate the 1 - β = 1.00, which was greater than the original 1 - β of 0.95 and indicating that the sample size of the study meets the sample size required for the mediation analysis. In addition, participants were recruited from seven tertiary grade-A hospitals of Tianjin city in Northern China through online questionnaires, and collected more questionnaires to ensure the generalization and universality of the research results, so as to minimize the deviation of the results caused by geography and hospital nature. In this way, included 1,058 participants were necessary and would greatly enhance the transparency and understanding of the study design, especially in relation to the mediation analysis. ”

And all the changes have been marked in red in the revised draft.

Last but not least, we have studied your comments point by point, we tried our best to improve the manuscript and made some changes to the manuscript. These changes will not influence the content and framework of the paper. And here we did not list the changes in detail but marked in red in the revised paper. We appreciate for your warm work earnestly and hope that the correction will meet with approval. All authors have approved the response letter and the revised version of the manuscript.

And due to our writing ability, understanding and logic are limited and we look forward to your generosity. Due to the time limit of our project and the need to employ professional title, and this paper is very important to our team, so we sincerely hope that you can recommend our revised version of the manuscript to accept for publication in the journal *Plos One* as soon as possible. If you have any queries, please do not hesitate to contact me.

Thank you again for your valuable comments and suggestions. I'm looking forward to hearing from you soon in due course.

Best regards,

Zhangyi Wang, Master's degree, Nurse Practitioner, [283537548@qq.com,](mailto:283537548@qq.com,)

Nursing Department, Affiliated Hengyang Hospital of Hunan Normal University & Hengyang Central Hospital, Hengyang City, Hunan Province, 421001, China.
